# Clinical and Surgical Outcomes in Patients with Lumbar Spine Pathologies: A Retrospective Study

**DOI:** 10.3390/reports9010079

**Published:** 2026-03-09

**Authors:** Adrian-Valentin Enache, Antonio-Daniel Corlatescu, Horia Petre Costin, Alexandru Vlad Ciurea

**Affiliations:** 1 Doctoral School, “Carol Davila” University of Medicine and Pharmacy, 050474 Bucharest, Romania; 2 Faculty of Medicine, “Carol Davila” University of Medicine and Pharmacy, 050474 Bucharest, Romania; 3 Sanador Clinical Hospital, 011033 Bucharest, Romania

**Keywords:** lumbar spine surgery, degenerative spondylolisthesis, spinal instrumentation, length of stay, neurological outcome, retrospective cohort, accelerated recovery, geriatric surgery

## Abstract

Background: Enhanced recovery pathways and modern fixation systems have shortened admission after lumbar spine surgery, yet the interplay between implant choice, comorbidity, and early morbidity remains incompletely defined. Methods: We undertook a retrospective, single-center cohort study of lumbar procedures performed at SANADOR Clinical Hospital (Bucharest, Romania) between 1 January 2023 and 31 May 2024. Eighty-six adult patients (64 women, 22 men; mean age 64.9 ± 10.8 years) met the inclusion criteria. Outcomes included length of stay (LOS), early postoperative neurological change (Frankel/American Spinal Injury Association (ASIA) Impairment Scale), and unplanned reoperation within 90 days. Analyses were performed in Python 3.11 (pandas, SciPy, statsmodels) and verified in IBM SPSS 28.0; α = 0.05. Results: Spondylolisthesis was the predominant diagnosis (60.5%), followed by lumbar stenosis (17.4%). Instrumentation was used in 75 cases (87.2%). Median LOS was 3 days (mean 3.8 ± 2.1), and most patients were discharged by postoperative day 4. LOS did not differ by interbody cage status (Mann–Whitney *p* = 0.459; median 3 vs. 3 days). Early postoperative neurological change occurred in 34.9% but improved or resolved in all cases by discharge; no permanent motor deficits were observed. Unplanned reoperation within 90 days occurred in 17.6%. In multivariable logistic regression for prolonged hospitalization (LOS > 4 days), early postoperative neurological change was associated with increased odds of prolonged LOS (OR 4.45, 95% CI 1.29–15.43; *p* = 0.018), whereas age showed only a borderline association (OR 1.06 per year, 95% CI 1.00–1.14; *p* = 0.065). Conclusions: In this single-center retrospective cohort, postoperative hospitalization was generally short. Prolonged LOS was more closely associated with early postoperative neurological change than with baseline comorbidity or interbody cage use. These findings should be interpreted as short-term, context-specific observations from a complex, predominantly instrumented referral cohort.

## 1. Introduction

Although the concept of spinal stability appears intuitive, it lacked a clear and widely accepted definition for many years. For example, spinal instability was initially described as excessive motion of the weight-bearing spine, inability to maintain normal anatomical alignment, and discordance between imaging findings and clinical presentation [1]. Even in the presence of these definitions, the evaluation of instability was based on a subjective interpretation of static spine imaging.

Low back pain is the world’s leading cause of disability, affecting an estimated 619 million people in 2020 and projected to reach 843 million by 2050, with a mean global prevalence ranging from 8% to 31% (reported as high as ~47% in some studies), varying by age, sex, and region, and disproportionately affecting students in academically demanding fields such as medicine [2]. Primary causes include trauma, tumors, infections, lumbar spondylolistesis, spinal stenosis, disk herniation, spinal deformity and especially lumbar instability [3]. Lumbar instability has long been hypothesized to contribute to low back pain and is frequently cited as a rationale for fusion; however, recent normative dynamic-radiography data suggest that commonly used radiographic “instability” thresholds may classify a substantial proportion of minimally symptomatic healthy individuals as unstable, underscoring the need for validated, pathology-specific criteria before instability is used as a stand-alone surgical justification [4]. Radiographic lumbar “instability” has historically been defined using flexion–extension radiographs and relatively simple motion measures, most commonly intervertebral rotation and sagittal translation. However, contemporary work emphasizes that no adequately validated, uniform diagnostic test exists, and newer approaches increasingly focus on standardized, rotation-adjusted metrics to better distinguish physiologic from pathologic motion [5,6].

Spondylolysis represents a defect in the pars interarticularis that typically results from acute trauma or chronic hyperextension of the spine and can lead to spondylolisthesis if vertebral translation occurs [7]. Spondylolysis can occur across all age groups; in children, it most often reflects repetitive micro-trauma, whereas in adults, it is more commonly associated with progressive degeneration of the ligamentous and facet joint complex [8]. In pediatric populations, spondylolysis is relatively common, affecting roughly ~5% of children and occurring most often at L5, and a meaningful minority subsequently develops isthmic spondylolisthesis, with progression risk influenced by factors such as bilaterality and age-related mechanical loading [9,10]. In adults, approximately 6–17% in global population develops degenerative spondylolisthesis, although most of these individuals are asymptomatic. Other types of spondylolisthesis include traumatic spondylolisthesis, resulting from high-energy injury and dysplastic spondylolisthesis, resulting from congenital anomalies [11]. Conservative treatment is typically recommended for spondylolisthesis, except for patients with neurological symptoms. Disease progression can stabilize over time, sometimes improving symptoms, and repetitive stress can also lead to further vertebral translation and subsequent nerve compression [12].

Spinal stenosis refers to the narrowing of the spinal canal, neural foramina, or lateral recesses, which leads to compression of the spinal cord or nerve roots and can produce a variety of clinical manifestations, including pain, numbness, and weakness in the lower extremities. Cervical and lumbar regions are most frequently affected [13,14,15]. Spinal stenosis can result from a variety of congenital and acquired conditions, including achondroplasia, trauma, disc herniation, ligament hypertrophy, spondylolisthesis, and complications from spine surgery [16,17]. However, only a small percentage of individuals with spinal pain develop clinically significant nerve compression. Additionally, degenerative changes are the most common etiology of spinal stenosis, particularly in patients older than 50 years, and spinal stenosis is the most common indication for spine surgery in patients older than 65 years. A decrease in lumbar lordosis can increase the likelihood of nerve root stenosis. Disc bulge, ligament thickening, or facet joint hypertrophy may contribute to stenosis and could ultimately lead to myelopathy or cauda equina syndrome. Most individuals with spinal stenosis will respond to conservative therapy; however, some will require surgical intervention [18,19]. This retrospective study aimed to describe short-term perioperative outcomes after lumbar surgery in a cohort in which degenerative disease predominated, with particular attention to length of stay, early postoperative neurological change, and 90-day unplanned reoperation, and to explore predictors of prolonged hospitalization.

## 2. Materials and Methods

### 2.1. Study Design and Setting

A single-center, retrospective cohort study was conducted at SANADOR Clinical Hospital, Bucharest, Romania. The study included all consecutive lumbar spine surgeries performed between 1 January 2023 and 31 May 2024.

### 2.2. Eligibility Criteria

Adult patients (≥18 years) were included if they underwent a lumbar or lumbosacral surgical procedure performed by the study surgical team at SANADOR Clinical Hospital between 1 January 2023 and 31 May 2024 for a primary lumbar spine pathology, including degenerative disease (e.g., spondylolisthesis/instability and lumbar stenosis), osteoporotic/low-energy vertebral fractures, spinal infection (discitis/osteomyelitis), spinal tumors, or symptomatic disc pathology requiring operative management. Patients were excluded if surgery was performed for acute high-energy trauma without an underlying degenerative substrate, if the index pathology was outside the lumbar/lumbosacral region, if prior lumbar instrumentation had been performed elsewhere (precluding standardized operative and perioperative data capture), or if records were incomplete such that prespecified inpatient outcomes and 90-day reintervention status could not be ascertained. The study was conducted within a private referral clinic; the cohort was enriched for more complex, instrumented cases and may not reflect the full spectrum of lower-acuity degenerative lumbar surgery seen in general practice.

### 2.3. Data Extracted

Extracted variables included demographic characteristics (age, sex, body mass index, obesity, diabetes), diagnosis and imaging data, surgical variables, and postoperative outcomes including postoperative neurologic status (preoperative Frankel and ASIA grades and at discharge), perioperative complications (wound, dural tear, thromboembolic event), length of stay (LOS), 30- and 90-day readmissions/reinterventions, and radiologic fusion at first-year followup when available. With diabetes prevalence of 9.3% (*n* = 8) and obesity prevalence of 33.7% (*n* = 29), power to detect moderate subgroup effects is limited (approximately ~15–20% for diabetes and ~40–50% for obesity), and thus clinically relevant associations cannot be excluded.

### 2.4. Statistical Analysis

All analyses were conducted using a prespecified Python 3.11.9 workflow (NumPy 1.26.4/Pandas 2.3.1, SciPy 1.15.3, Statsmodels 0.14.5, and scikit-learn 1.6.1). Continuous variables are reported as mean ± standard deviation (SD) and median with interquartile range (IQR), together with minimum and maximum values. Categorical variables are summarized as counts and percentages. All tests were two-sided and interpreted at a nominal significance threshold of α = 0.05.

#### 2.4.1. Data Preparation, Missing Data, and Assumptions

The analyses were performed on the cleaned dataset exported as a comma-separated file. Variable coding followed clinically interpretable contrasts: unplanned reintervention within 90 days was analyzed as a binary outcome (0/1), sex was coded as male vs. female, and cage material was operationalized as polyetheretherketone (PEEK) vs. non-PEEK to avoid sparse multi-category modeling. Missing data were handled by complete-case analysis within each test/model (i.e., observations with missing values in the variables required for a given analysis were excluded only from that analysis). For the multivariable logistic regression, only patients with complete data for all modeled predictors and the outcome were included; the final number of observations and number of events were reported alongside the model results.

Normality of continuous variables was evaluated using the Shapiro–Wilk test when group size permitted (*n* ≤ 50). Given the limited interpretability of the Shapiro–Wilk at larger sample sizes and its sensitivity to minor deviations, the Shapiro–Wilk was not applied when *n* exceeded this threshold. Where appropriate, distributional assumptions were therefore addressed by using robust/non-parametric methods and by reporting both parametric and non-parametric summary statistics.

#### 2.4.2. Univariable Comparisons and Effect Size Reporting

For two-group comparisons of continuous outcomes, the inferential test was selected according to distributional compatibility. When both groups were compatible with normality, independent-samples *t*-tests were used with variance handling based on Levene’s test (Student’s *t*-test if equal variances; Welch’s *t*-test otherwise). When normality assumptions were not met, Mann–Whitney U tests were applied. Effect sizes were reported as Cohen’s d for parametric comparisons and rank-biserial correlation for Mann–Whitney tests, alongside group means or medians, as appropriate.

Differences in LOS across diagnostic categories were assessed using the Kruskal–Wallis test.

For categorical variables, associations were examined using Pearson’s chi-square test. When a 2 × 2 table contained expected cell counts < 5, Fisher’s exact test was used instead. Effect size was reported as φ for 2 × 2 chi-square tests or Cramér’s V for larger contingency tables. For Fisher’s exact tests, odds ratios with confidence intervals were computed from the 2 × 2 contingency table.

Monotonic associations between continuous variables (e.g., age and length of stay) were assessed using Spearman’s rank correlation (ρ) with complete-pair handling.

##### Subgroup Analyses with Confidence Intervals

For predefined subgroup comparisons (e.g., diabetes: yes vs. no; obesity: yes vs. no), we generated a standardized summary table reporting both central estimates and uncertainty. Length of stay (LOS) was summarized within each subgroup as mean ± SD, together with 95% confidence intervals (CI) for the mean computed using the t-distribution. Between-group LOS differences were tested using the Welch independent-samples *t*-test, selected a priori to remain valid under unequal variances. Early postoperative neurological change frequency was reported as a proportion within each subgroup, with 95% CIs calculated using the Wilson score method. Associations between subgroup membership and early postoperative neurological change (both binary) were evaluated using Fisher’s exact test when expected cell counts were <5, and otherwise using Pearson’s chi-square test.

#### 2.4.3. Multivariable Modeling Strategy

To identify independent predictors of unplanned reintervention within 90 days, a multivariable logistic regression model was fitted with unplanned reintervention within 90 days (0/1) as the dependent variable. Predictors were entered simultaneously (forced-entry), rather than by stepwise selection, to preserve clinical interpretability and to limit optimism associated with data-driven variable selection. The prespecified covariate set included age, sex (male), cage material (PEEK), obesity, diabetes mellitus, and early postoperative neurological change. Regression coefficients were exponentiated and reported as odds ratios (ORs) with 95% confidence intervals and two-sided *p*-values.

Sample size justification. The cohort size (*n* = 86) reflected consecutive eligible cases treated within the defined study period at a single center; therefore, the sample size was determined by case availability rather than an a priori power calculation. Accordingly, multivariable findings are interpreted as exploratory, with emphasis placed on effect estimates (ORs and confidence intervals), model performance metrics, and clinical plausibility, rather than on statistical significance alone. The number of unplanned reintervention within 90 days events included in the regression was reported, and model limitations related to sample size and events-per-predictor were addressed in the discussion. The modest cohort size reflects the fact that the analysis was limited to a single surgical team within a private referral clinic, and therefore does not represent the full spectrum of degenerative lumbar surgery seen in general practice. This practice setting is enriched for more complex presentations, which likely contributes to a higher proportion of instrumented procedures than in unselected degenerative stenosis cohorts. Accordingly, our findings should be interpreted as describing short-term outcomes in a complex degenerative surgical population, and may not be generalizable to lower-acuity degenerative cases treated in broader institutional settings.

Multicollinearity was assessed using variance inflation factors (VIF) and was low (VIF ~1.03–1.21). Calibration was evaluated with the Hosmer–Lemeshow test (χ^2^ = 12.46, df = 8, *p* = 0.132), showing no evidence of poor fit. Discrimination was quantified using ROC-AUC (0.745). Explained variation was summarized using pseudo-R^2^ indices (Cox–Snell R^2^ = 0.125; Nagelkerke R^2^ = 0.206), consistent with modest explanatory power in this multifactorial endpoint. This level of explained variation is consistent with the multifactorial nature of unplanned reintervention within 90 days and the likelihood of unmeasured contributors (e.g., baseline disease severity, imaging characteristics, operative complexity, postoperative management, and follow-up heterogeneity), which limits the model’s utility as a comprehensive predictor and supports interpretation primarily as an association model.

In addition, a prespecified logistic regression model was fitted for prolonged hospitalization defined as LOS > 4 days, using the same covariate set; ORs with 95% CIs, ROC-AUC, VIF, and Hosmer–Lemeshow calibration were reported.

#### 2.4.4. Outliers and Sensitivity Analyses

Length of stay demonstrated a high-end observation (16 days). Outliers were not automatically excluded from primary analyses. To evaluate robustness, a prespecified sensitivity analysis excluded LOS ≥ 16 and repeated the LOS-by-cage comparison; results were materially unchanged (Mann–Whitney U = 918.5, *p* = 0.554; median LOS 3 vs. 3 days), indicating that inferences were not driven by this extreme value.

#### 2.4.5. Reporting and Reproducibility

All statistical outputs (descriptive summaries, univariable tests, and multivariable model results including diagnostics) were exported to spreadsheet format with frozen headers to facilitate auditability. Figures were generated using Matplotlib 3.9.4. The full analysis script and the cleaned dataset structure allow independent reproduction of all reported results.

### 2.5. Ethical Approval

This retrospective study was reviewed and approved by the Ethics Committee for Clinical Studies Approval of SANADOR Clinical Hospital, Bucharest, Romania, which issued a favorable opinion on 27 March 2023. In our institution, the ethics review pathway for retrospective studies is documented through this formal favorable opinion and does not generate a separate numeric approval code. The study was conducted in accordance with the principles of the Declaration of Helsinki. Given the retrospective design, minimal risk to participants, and the impracticability of obtaining informed consent from all eligible patients during the study period, the Ethics Committee approved a waiver of informed consent. Patient data were handled in accordance with institutional confidentiality procedures and applicable GDPR requirements; data were extracted from hospital records, de-identified prior to analysis, and accessible only to the study team.

### 2.6. Surgical Technique

Interbody fusion was used selectively when disc height restoration/anterior support was needed, most commonly in cases with substantial disc collapse and foraminal stenosis, revision surgery, or alignment objectives; posterolateral fusion alone was performed when stability could be achieved without disc space work. Decompression was defined as posterior neural decompression tailored to pathology and included laminotomy/hemilaminectomy or laminectomy with medial facetectomy and/or foraminotomy, with “over-the-top” contralateral decompression used when bilateral decompression was achieved through a unilateral approach.

## 3. Results

A total of 86 consecutive lumbar spine surgeries performed between 1 January 2023 and 31 May 2024 were included in the study.

### 3.1. Patient Demographics

The majority of the 86 patients included in this study were women (64), which gave the group a female-to-male ratio of 2.9:1.

**Figure 1:**
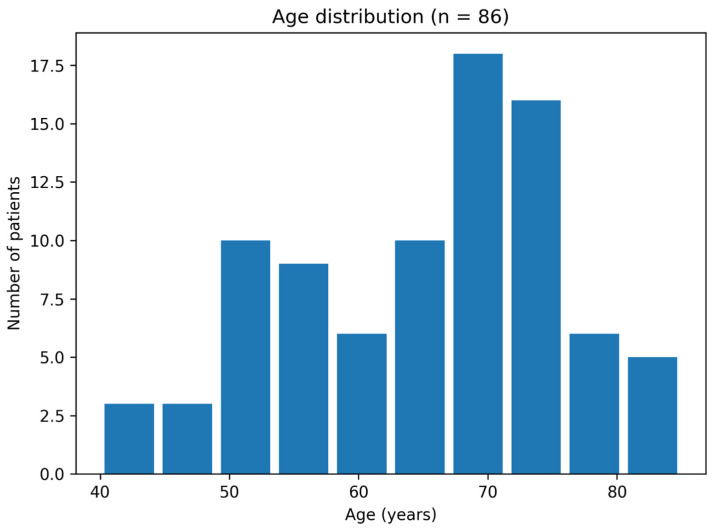
Age distribution in our study cohort.

The average age of the subjects included in this study was 64.9 ± 10.8 years with an approximate normal age distribution, a median age of 67.5 years (Interquartile Range [IQR] = 18), and a full age range of 40–85 years. Obese individuals made up 29 of the patients (33.7%), while diabetic mellitus was reported in eight (9.3%). Age showed a weak positive association with length of stay on Spearman correlation (ρ = 0.271, *p* = 0.012). There was no significant gender-based age difference detected (t = 1.14, df = 84, *p* = 0.26) as seen in Figure 1.

### 3.2. Distribution of Diagnoses

**Figure 2:**
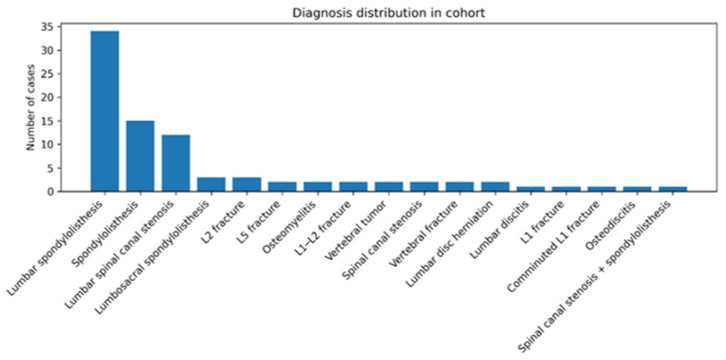
Distribution of diagnoses in our study group.

Degenerative spine conditions represented the greatest proportion of the diagnoses, as shown in Figure 2. Spondylolisthesis was diagnosed in 52 of the patients (60.5%), lumbar spinal stenosis in 15 (17.4%) and osteoporotic fractures/low-energy fractures in 11 (12.8%). Discitis/osteomyelitis (*n* = 4, 4.7%), Spinal tumors (*n* = 2, 2.3%), and repair of an isolated disc herniation using instrumentation (*n* = 2, 2.3%) were less common. Length of stay differed across diagnostic categories (Kruskal–Wallis H = 11.847, *p* = 0.037). Descriptively, median LOS was higher in the infection and tumor subgroups (both 6.5 days) and lowest in degenerative spondylolisthesis (3.0 days), noting the small sample sizes in the non-degenerative groups.

### 3.3. Surgical Intervention Types and Instrumentation Use

**Figure 3:**
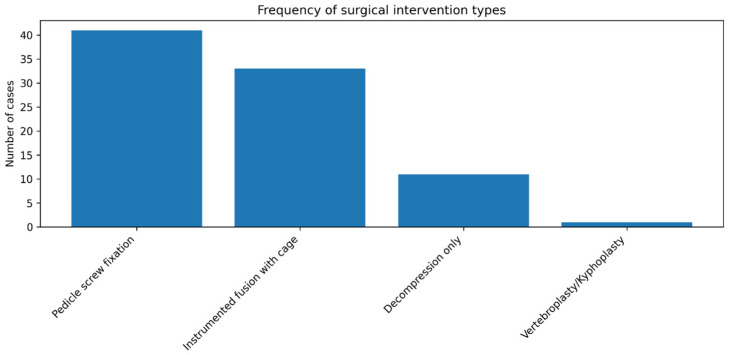
Frequency of the surgical intervention types.

Four main categories of surgical intervention were identified (Figure 3). Pedicle screw constructs without an interbody graft were the most commonly performed procedure (*n* = 41, 47.7%). A circumferential fusion using a polyetheretherketone (PEEK) cage was performed in 33 patients (38.4%), while decompression without implants was chosen for 11 patients (12.8%); one vertebro-/kyphoplasty completed the series.

**Figure 4:**
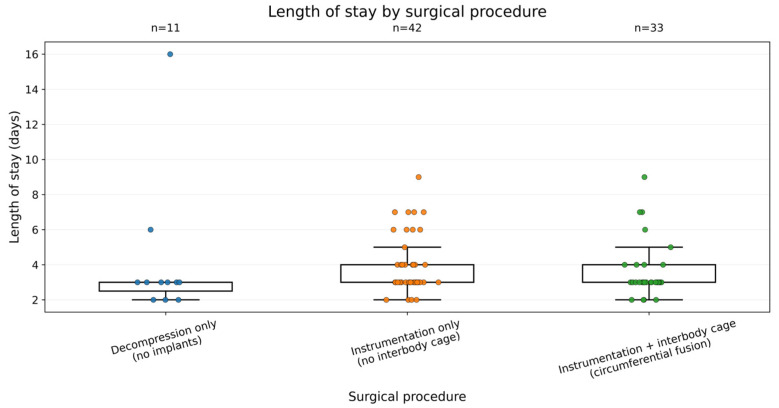
Length of stay by surgical procedure. Procedure type (decompression, instrumentation, and interbody fusion) was selected based on pathology severity, instability, and alignment objectives. Comparisons of outcomes across procedure categories should be interpreted as descriptive and are subject to confounding by indication.

Instrumentation was used in 75 of the 86 operations (87.2%). A significant association was observed between the presence of mechanical instability prior to surgery (recorded in 70 of the patients) and the use of instrumentation (instrumented segments: 94% vs. non-instrumented segments: 69%; χ^2^ = 5.29, *p* < 0.02). However, instrumentation did not result in increased hospitalization time. Length of stay did not differ between instrumented and non-instrumented procedures (Mann–Whitney U = 505.5, *p* = 0.192; median 3 vs. 3 days; *n* = 75 vs. 11). Length of stay did not differ by interbody cage status (Mann–Whitney U = 951.5, *p* = 0.459; median 3 vs. 3 days) Figure 4.

### 3.4. Post-Operative Outcomes

**Figure 5:**
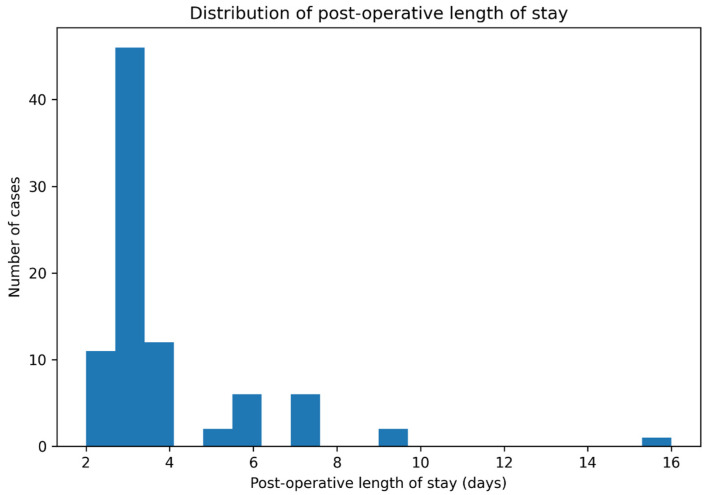
Distribution of post-operative length of stay.

Thirty (34.9%) of the 86 evaluable patients experienced transient neurologic decline post-operatively. All of the declines were classified as Frankel/ASIA grades C–D and had resolved to some degree by discharge. None of the patients left the hospital with a new permanent motor deficit. The LOS exhibited a positively skewed distribution (see Figure 5) with a mean of 3.8 ± 2.1 days, a median of 3 days (IQR = 1), and a single extreme value of 16 days (due to comorbid pneumonia). Approximately 84% of the patients were discharged on or before post-operative day 4.

Fifteen patients (17.6%, 15/85 with available follow-up) required unplanned reintervention within 90 days. This outcome reflects early postoperative reinterventions within a short follow-up horizon and is reported descriptively without comparison to long-term adjacent segment disease (ASD) rates.

The relatively high rate of transient postoperative neurological changes observed in our cohort may be explained, at least in part, by case-mix and by outcome definition. Our study population predominantly comprised patients with radiographic and clinical instability requiring instrumented fixation, often accompanied by advanced degenerative stenosis, spondylolisthesis, and multilevel pathology, conditions that typically necessitate wider decompression and increased nerve-root manipulation, with a consequent propensity for short-lived sensory changes or transient radicular symptom exacerbations. In parallel, direct comparison with pooled incidences from systematic reviews is inherently limited because our definition captured any deviation from baseline on early postoperative examination, including minor sensory fluctuations and pain-predominant radicular exacerbations, whereas many studies restrict “neurological deficit” to new objective motor weakness. This broader ascertainment may inflate the apparent rate while improving sensitivity for transient neuropraxia or irritative phenomena; importantly, no neurological changes persisted at discharge, supporting a predominantly reversible mechanism rather than permanent iatrogenic injury.

### 3.5. Sub-Group Analyses

In predefined subgroup analyses, we observed no statistically significant differences in length of stay (LOS) or early postoperative neurological change according to diabetes or obesity status. Mean LOS was comparable in patients with versus without diabetes (3.9 ± 1.9 vs. 3.8 ± 2.1 days; Welch *t*-test *p* = 0.954) and in patients with versus without obesity (4.1 ± 2.7 vs. 3.7 ± 1.6 days; Welch *t*-test *p* = 0.417). The frequency of immediate postoperative neurological change was similarly not different by diabetes status (37.5% vs. 34.6%; Fisher’s exact *p* = 1.000) or by obesity status (31.0% vs. 36.8%; χ^2^ *p* = 0.593). Given the small diabetes subgroup (*n* = 8) and the modest obesity subgroup size (*n* = 29), these analyses were underpowered; therefore, non-significant findings reflect limited power and should not rule out clinically meaningful effects (Table 1 and Table 2).

### 3.6. Additional Observations

Reliance upon fixation hardware was not associated with longer hospitalization in univariable analyses. In the adjusted LOS > 4 days model, early postoperative neurological change was associated with increased odds of prolonged stay, whereas age showed only a borderline association.

LOS also showed variation across diagnostic categories in univariable analysis (Kruskal–Wallis *p* = 0.037), with descriptively longer stays in infection and tumor cases.

### 3.7. Multivariable Analysis of Prolonged Hospitalization (LOS > 4 Days)

A multivariable logistic regression model was fitted for prolonged hospitalization (LOS > 4 days) in the complete-case dataset (*n* = 78; events = 16). Early postoperative neurological change was independently associated with prolonged LOS (OR 4.45, 95% CI 1.29–15.43; *p* = 0.018). Age showed a borderline association (OR 1.06 per year, 95% CI 1.00–1.14; *p* = 0.065), while sex, obesity, diabetes, and PEEK cage use were not significant predictors. Model calibration was acceptable (Hosmer–Lemeshow *p* = 0.896) and discrimination was moderate (AUC = 0.768); collinearity was low (VIF range 1.02–1.22).

## 4. Discussion

This study provides a detailed description of current trends in lumbar spine surgery at a high-volume center in Eastern Europe, in the context of international evidence. Three conclusions emerged: (i) female preponderance and a case-mix in which degenerative conditions predominated, with smaller proportions of fracture, infection, and tumor cases, (ii) an extensive reliance on pedicle screw constructs without increasing length of stay and (iii) short LOS overall, with prolonged hospitalization more strongly associated with early postoperative neurological change than with baseline comorbidity or implant selection.

There were significantly more women in the sample (74.4%) compared to previously reported figures from large U.S. based databases (56–61%) of degenerative spondylolisthesis and stenosis [20]. The female predominance in our cohort (74.4%) needs to be taken into consideration. Sex distributions in degenerative lumbar surgery vary by region and indication, and our case-mix, largely patients with degenerative instability requiring fixation, often associated with spondylolisthesis and advanced segmental degeneration, may have enriched for conditions more frequently treated surgically in women in some populations. Local referral pathways and practice patterns, together with the single-center nature of the study, may further accentuate this demographic skew. Accordingly, this distribution likely reflects regional epidemiology and selection and should be considered when extrapolating our findings. Low-grade spondylolisthesis (60.5%) was the predominant diagnosis, consistent with the same U.S. database in which fusion for spondylolisthesis surpassed decompression alone as a procedure in 2016 and currently accounts for greater than 50% of all spinal surgeries performed. The fixation preference of this institution is also demonstrated by the high instrumentation rate of 87%, which is greater than the 49% fusion utilization reported in 162,000 American patients; however, the effect of this preference on hospitalization resource use was negligible, as there was no difference in the length of hospital stay between instrumented and non-instrumented decompressions [20]. The median length of stay (LOS) of three days (mean 3.8 ± 2.1) compares favourably with most contemporary series. A Chinese single-centre cohort of 310 lumbar fusions reported a median LOS of six days [21], while minimally invasive TLIF studies typically cite means of 3–4 days, but can go up to 7 days according to some studies [22,23] and large U.S. registry work places national averages at 3.7–4.0 days [24]. Thus, the enhanced recovery pathway adopted at our clinic appears to deliver discharge nearly 24 h earlier than comparable institutions. In the multivariable model for prolonged hospitalization (LOS > 4 days), early postoperative neurological change was associated with increased odds of prolonged LOS (OR 4.45; *p* = 0.018), whereas age showed only a borderline association after adjustment (OR 1.06 per year; *p* = 0.065). This association likely reflects clinical caution, extended postoperative monitoring, and additional rehabilitation needs in patients with early neurological change. Although age did not reach conventional significance in our adjusted LOS > 4 days model, the direction of effect was consistent with prior reports in which older age tended to be associated with longer hospitalization such as the findings in an instrumented spine study of 706 patients where age > 75 years independently prolonged LOS by 0.6 days [24] and from a Chinese nomogram study where age ≥ 65 was overrepresented in the prolonged-LOS quartile [21]. However, both diabetes and obesity, two robust predictors of LOS in multiple North-American studies [24], did not have a statistically significant effect in this sample. Notably, the age effect was attenuated after adjustment relative to the univariable association, suggesting that part of the apparent age-related increase in LOS may be mediated or confounded by early postoperative neurological change and related clinical management. It is possible that the tighter perioperative metabolic control achieved through the enhanced recovery protocol (ERP) at our clinic, combined with the majority of the patients undergoing single-level constructs, contributed to the lack of significance of diabetes and obesity in this study. Although we did not detect statistically significant associations between diabetes/obesity and early outcomes, the small subgroup sizes, especially diabetes, substantially limit statistical power; therefore, these findings should be interpreted as failure to detect an effect rather than evidence that no effect exists. Postoperative transient neurologic deficits occurred in 34.9% of the sample, which is significantly higher than the pooled incidence of 5.7–9% in a systematic review of 2783 patients [25]. There were likely two reasons why the incidence in this study was so much higher than in the systematic review: (1) our definition of neurologic deficits included any grade change on the five-point Frankel/ASIA scale rather than only motor loss, and (2) we assessed patients on the first postoperative day, possibly uncovering some minor sensory changes that might have been underreported in other studies. Importantly, all patients who experienced any neurologic deficit had either resolution or improvement of their deficits prior to discharge, which is consistent with the low rate of permanent injury (<2%) reported in the systematic review [25].

Our 90-day unplanned reintervention rate should not be compared with published 5-year adjacent segment disease (ASD) rates, because the time horizon and outcome definition differ substantially: ASD is a long-term degenerative phenomenon that typically evolves over years, whereas our endpoint captures early postoperative reinterventions. Interpreted appropriately, the observed early reintervention burden warrants careful consideration and may reflect a combination of case complexity and early postoperative events, rather than long-term adjacent segment degeneration. Future studies with longer follow-up are necessary to quantify true ASD incidence and longer-term reintervention risk. However, the rate of unplanned reintervention within 90 days in this study (17.4%) is comparable with the rate of early reintervention (16.1%) reported in a recent study of 4671 revision fusions at a single US-based center. The profile of indications for reintervention in this study, adjacent segment disease (40%), hardware prominence (27%), and residual stenosis (20%), is consistent with the profiles reported in both studies and reinforces the idea that the majority of early failures are due to biologic rather than mechanical causes [26]. A biologic explanation for early reintervention is further supported by contemporary evidence showing that patient- and tissue-level factors, rather than construct mechanics alone, often dominate the early failure phenotype after lumbar fusion. In a large cohort study evaluating early versus late reintervention after lumbar fusion, the authors reported that early reinterventions clustered around early postoperative complications and were associated with host factors such as osteoporosis and diabetes, whereas ASD emerged more prominently as a later driver of reintervention over longer follow-up [27]. Complementing this, a more recent Spine Journal case–control study focused on adjacent segment degeneration requiring revision emphasized that “early” revision for ASD is not simply a time-compressed mechanical complication, but reflects a confluence of baseline degenerative burden, biologic susceptibility, and clinical context, underscoring why early reintervention patterns should be interpreted through a biologic lens [28]. The results of this study suggest that the combination of a pro-active fixation strategy with an aggressive mobilization protocol can result in discharge by the third postoperative day without an excessive amount of neurologic or infectious morbidity. Accordingly, strategies that minimize transient neurological irritation and standardize postoperative monitoring and mobilization pathways may help reduce prolonged hospitalization; age may contribute to risk, but did not reach conventional significance after adjustment in our model. Similarly, neither obesity nor controlled diabetes should preclude instrumentation when biomechanically necessary. Lastly, the relative frequency of early revision emphasizes the need for long-term surveillance of adjacent levels, especially in patients undergoing fusion for L4–L5 spondylolisthesis.

Our rate of immediate postoperative neurological change appears higher than pooled incidences reported in systematic reviews; however, direct comparison is limited by differences in definitions and ascertainment. Many studies restrict “neurological deficit” to a new objective motor deficit, whereas our endpoint captured any deviation from baseline on early postoperative examination, including transient sensory fluctuations and pain-predominant radicular exacerbations, which likely inflates the apparent incidence. In addition, our cohort was enriched for patients with degenerative instability requiring instrumented fixation, often with advanced stenosis, spondylolisthesis, and multilevel disease, potentially necessitating wider decompression and greater nerve-root handling and thereby increasing transient sensory/radicular changes. Importantly, the neurological changes observed were transient and resolved by discharge, consistent with reversible mechanisms such as nerve-root irritation or edema-related neuropraxia. Since assessments were confined to the inpatient period, these findings reflect short-term postoperative neurological dynamics, and future prospective studies with standardized deficit definitions and longer follow-up are needed for more direct comparability.

### Limitations

This study has several limitations that should be considered when interpreting the findings. First, the retrospective design relies on the completeness and accuracy of routine clinical documentation and is therefore susceptible to information bias and residual confounding. Second, a critical limitation is the absence of validated patient-reported outcome measures (PROMs), such as the Oswestry Disability Index (ODI), Visual Analog Scale (VAS) for pain, or health-related quality-of-life instruments; consequently, patient-centered functional outcomes (pain relief, disability, and quality of life) and the functional effectiveness of surgery cannot be assessed. Third, the cohort size (*n* = 86) limits precision, and subgroup analyses, particularly for smaller comorbidity strata (e.g., diabetes), are underpowered; thus, non-significant findings should be interpreted as an inability to detect an association rather than proof that no effect exists. Fourth, this was a single-center study, which may reflect institution-specific pathways, perioperative monitoring intensity, and case-mix, thereby limiting external validity. Fifth, follow-up was restricted to 90 days, which is appropriate for early postoperative events but insufficient to evaluate longer-term outcomes such as adjacent segment disease, late complications, or durability of symptom control. Finally, while multivariable modeling was performed to identify predictors of prolonged hospitalization, the explained variance was modest, and important unmeasured confounders (e.g., smoking status, ASA class, operative duration, number of operated levels, and detailed radiographic severity) may have influenced length of stay and early postoperative events; longer, multicenter, prospectively designed studies incorporating PROMs and standardized outcome definitions are warranted to confirm and extend these findings. Because follow-up was limited to 90 days, our study is insufficient to assess adjacent segment disease, which manifests over years, and we therefore cannot draw conclusions regarding long-term ASD incidence or durability of surgical outcomes.

Subgroup analyses were underpowered (diabetes *n* = 8; obesity *n* = 29), yielding inadequate statistical power; therefore, our null findings should be interpreted as failing to detect an effect rather than evidence that no effect exists. Furthermore, because this private referral cohort was enriched for complex and instrumented cases, the findings may not generalise to lower-acuity or non-instrumented degenerative lumbar cases.

## 5. Conclusions

In this single-center retrospective cohort of patients undergoing surgery for degenerative lumbar pathology, predominantly complex cases managed with instrumented fixation, postoperative hospitalization was short overall, with a median length of stay of three days. In multivariable analysis of prolonged hospitalization (LOS > 4 days), early postoperative neurological change was associated with increased odds of prolonged LOS, while age showed only a borderline association; sex, obesity, diabetes, and PEEK cage use were not significant predictors. Early postoperative neurological changes were observed under a broad ascertainment approach and generally improved by discharge; however, their occurrence underscores the need for cautious interpretation of perioperative safety signals in this cohort. Unplanned reintervention within 90 days occurred in a meaningful proportion of patients and should be interpreted strictly as an early postoperative endpoint rather than as evidence regarding long-term adjacent segment disease or durability, which requires extended follow-up. Because outcomes were limited to short-term measures and treatment selection was influenced by case complexity, these findings should be viewed as descriptive and hypothesis-generating rather than as evidence of the effectiveness or safety of any specific instrumentation strategy. Future prospective, preferably multicenter studies incorporating standardized outcome definitions, validated patient-reported outcomes, and longer follow-up are needed to clarify patient-centered benefit and long-term risks after surgery for degenerative lumbar disease.

## Figures and Tables

**Table 1:** Baseline patient characteristics, pathology distribution, operative level, and perioperative variables; ASA, American Society of Anesthesiologists physical status classification; IQR, interquartile range; PMMA, polymethylmethacrylate (bone cement); PEEK, polyetheretherketone; SD, standard deviation.

Characteristic	Overall (N = 86)
Age (years), median [IQR]	67.5 [55.0–73.0]
Age (years), mean ± SD	64.9 ± 10.8
Sex: Female	64 (74.4%)
Sex: Male	22 (25.6%)
Diagnosis	
Degenerative spondylolisthesis	52 (60.5%)
Lumbar spinal stenosis	15 (17.4%)
Fracture	11 (12.8%)
Infection (discitis/osteomyelitis)	4 (4.7%)
Disc herniation	2 (2.3%)
Tumor	2 (2.3%)
Mechanical instability (*n* = 83)	70 (84.3%)
Surgical level	
L4–L5	36 (41.9%)
L5–S1	13 (15.1%)
L3–L4	12 (14.0%)
L1–L2	5 (5.8%)
L3–L5	4 (4.7%)
L2–L3	3 (3.5%)
L2	3 (3.5%)
L1	2 (2.3%)
Other	8 (9.3%)
ASA class (*n* = 34)	
ASA 1	1 (2.9%)
ASA 2	2 (5.9%)
ASA 3	6 (17.6%)
ASA 4	25 (73.5%)
Obesity	29 (33.7%)
Diabetes	8 (9.3%)
Radicular irradiation	83 (96.5%)
Radicular irradiation pattern (among patients with irradiation)	
Bilateral	19 (22.9%)
Right	19 (22.9%)
Right > Left	18 (21.7%)
Left	15 (18.1%)
Left > Right	12 (14.5%)
Surgery/implants	
Instrumentation performed	75 (87.2%)
Cement augmentation/PMMA	1 (1.2%)
Interbody cage used	33 (38.4%)
Cage material (among cages)	
PEEK	33 (100.0%)
Multilevel involvement (≥3 levels) (*n* = 78)	9 (11.5%)
Length of stay (days), median [IQR]	3.0 [3.0–4.0]
Length of stay (days), mean ± SD	3.8 ± 2.1
Postoperative neurological change	30 (34.9%)
Unplanned reoperation ≤ 90 d (*n* = 85)	15 (17.6%)
Frankel grade (*n* = 48)	
B	1 (2.1%)
C	6 (12.5%)
D	37 (77.1%)
E	4 (8.3%)
Any comorbidity recorded (*n* = 19)	19 (100.0%)

**Table 2:** Table of sub-group analyses.

Sub-Group (Yes vs. No)	*n* (Yes)	*n* (No)	LOS Mean ± SD (Yes)	LOS Mean 95% CI (Yes)	LOS Mean ± SD (No)	LOS Mean 95% CI (No)	*p* (LOS) [Welch t]	Neurological-Deficit % (Yes)	Neuro-Deficit % 95% CI (Yes)	Neurological-Deficit % (No)	Neuro-Deficit % 95% CI (No)	*p* (Deficit) [Fisher]	*p* (Deficit) [Chi-Square]	*p* (LOS) Display	*p* (Deficit) Display
Diabetes	8	78	3.9 ± 1.9	2.3–5.5	3.8 ± 2.1	3.4–4.3	0.954331	37.5	13.7–69.4	34.6	25.0–45.7	1		0.954	1.000
Obesity	29	57	4.1 ± 2.7	3.1–5.2	3.7 ± 1.6	3.3–4.1	0.417374	31	17.3–49.2	36.8	25.5–49.8		0.593181	0.417	0.593

## Data Availability

Data supporting the findings are available from the corresponding author upon reasonable request, subject to institutional approval and compliance with applicable data protection regulations. Code is available upon reasonable request.

## References

[1] Knutsson F. (1944). The Instability Associated with Disk Degeneration in the Lumbar Spine. Acta Radiol..

[2] Behairy M., Odeh S., Alsourani J., Talic M., Alnachef S., Qazi S., Mazhar M.A., Tamim H. (2025). Prevalence of Lower Back Pain (LBP) and Its Associated Risk Factors Among Alfaisal University Medical Students in Riyadh, Saudi Arabia: A Cross-Sectional Study. Healthcare.

[3] Li N., Scofield J., Mangham P., Cooper J., Sherman W., Kaye A.D. (2022). Spondylolisthesis. Orthop. Rev..

[4] Suzuki M., Tanaka Y., Hashimoto K., Tsubakino T., Hoshikawa T., Takahashi K., Latt M.M., Aizawa T. (2024). Validating the Definition of Lumbar Instability—A Cross-Sectional Study with 420 Healthy Volunteers. J. Clin. Med..

[5] White A.A., Panjabi M.M. (1990). Clinical Biomechanics of the Spine.

[6] Hipp J.A., Reitman C.A., Buser Z., Chaput C.D., Ghogawala Z., Grieco T.F. (2025). Automated Radiographic Metrics for Diagnosing Lumbar Spine Instability: A Cross-Sectional Observational Study. Quant. Imaging Med. Surg..

[7] Debnath U.K. (2021). Lumbar Spondylolysis—Current Concepts Review. J. Clin. Orthop. Trauma.

[8] Madden V., Ayoub A., Thomas J., Thomas I. (2026). Spondylolysis: A Narrative Review of Etiology, Diagnosis, and Management. Int. J. Environ. Res. Public Health.

[9] Aoki Y., Takahashi H., Nakajima A., Kubota G., Watanabe A., Nakajima T., Eguchi Y., Orita S., Fukuchi H., Yanagawa N. (2020). Prevalence of Lumbar Spondylolysis and Spondylolisthesis in Patients with Degenerative Spinal Disease. Sci. Rep..

[10] Saremi A., Goyal K.K., Benzel E.C., Orr R.D. (2024). Evolution of Lumbar Degenerative Spondylolisthesis with Key Radiographic Features. Spine J..

[11] Soriano E., Bellinger E. (2020). Adult Degenerative Lumbar Spondylolisthesis: Nonoperative Treatment. Semin. Spine Surg..

[12] Shah S.A., Saller J. (2016). Evaluation and Diagnosis of Back Pain in Children and Adolescents. J. Am. Acad. Orthop. Surg..

[13] Tang C., Moser F.G., Reveille J., Bruckel J., Weisman M.H. (2019). Cauda Equina Syndrome in Ankylosing Spondylitis: Challenges in Diagnosis, Management, and Pathogenesis. J. Rheumatol..

[14] Glassman D.M., Magnusson E., Agel J., Bellabarba C., Bransford R.J. (2019). The Impact of Stenosis and Translation on Spinal Cord Injuries in Traumatic Cervical Facet Dislocations. Spine J..

[15] Bindal S., Bindal S.K., Bindal M., Bindal A.K. (2019). Noninstrumented Lumbar Fusion with Bone Morphogenetic Proteins for Spinal Stenosis with Spondylolisthesis in the Elderly. World Neurosurg..

[16] Messiah S., Tharian A.R., Candido K.D., Knezevic N.N. (2019). Neurogenic Claudication: A Review of Current Understanding and Treatment Options. Curr. Pain Headache Rep..

[17] Bagley C., MacAllister M., Dosselman L., Moreno J., Aoun S.G., El Ahmadieh T.Y. (2019). Current Concepts and Recent Advances in Understanding and Managing Lumbar Spine Stenosis. F1000Research.

[18] Akar E., Somay H. (2019). Comparative Morphometric Analysis of Congenital and Acquired Lumbar Spinal Stenosis. J. Clin. Neurosci..

[19] Melancia J.L., Francisco A.F., Antunes J.L. (2014). Spinal Stenosis. Handbook of Clinical Neurology.

[20] Ball J.R., Gallo M.C., Kebaish K., Hang N., Ton A., Hernandez F., Abdou M., Karakash W.J., Wang J.C., Hah R.J. (2024). National Trends in Lumbar Degenerative Spondylolisthesis With Stenosis Treated With Fusion Versus Decompression. Neurospine.

[21] Lu C.-X., Huang Z.-B., Chen X.-M., Wu X.-D. (2022). Predicting Prolonged Postoperative Length of Stay Risk in Patients Undergoing Lumbar Fusion Surgery: Development and Assessment of a Novel Predictive Nomogram. Front. Surg..

[22] Wang Y.-Y., Chung Y.-H., Huang C.-H., Hu M.-H. (2024). Comparison of Minimally Invasive Transforaminal Lumbar Interbody Fusion and Midline Lumbar Interbody Fusion in Patients with Spondylolisthesis. J. Orthop. Surg..

[23] Pradeep K., Pal B., Mukherjee K., Shetty G.M. (2025). Transforaminal Lumbar Interbody Fusion (TLIF) Surgery: A Finite Element Analysis of Open and Minimally Invasive Approach on L4-L5 Segment. Heliyon.

[24] Lundgren M.E., Detwiler A.N., Lamping J.W., Gael S.L., Chen N.-W., Kasir R., Whaley J.D., Park D.K. (2023). Effect of Instrumented Spine Surgery on Length of Stay. JAAOS Glob. Res. Rev..

[25] Ghobrial G.M., Williams K.A., Arnold P., Fehlings M., Harrop J.S. (2015). Iatrogenic Neurologic Deficit after Lumbar Spine Surgery: A Review. Clin. Neurol. Neurosurg..

[26] Lambrechts M.J., Toci G.R., Siegel N., Karamian B.A., Canseco J.A., Hilibrand A.S., Schroeder G.D., Vaccaro A.R., Kepler C.K. (2023). Revision Lumbar Fusions Have Higher Rates of Reintervention and Result in Worse Clinical Outcomes Compared to Primary Lumbar Fusions. Spine J..

[27] Wang S.-K., Wang P., Li X.-Y., Kong C., Niu J.-Y., Lu S.-B. (2022). Incidence and Risk Factors for Early and Late Reintervention Following Lumbar Fusion Surgery. J. Orthop. Surg..

[28] Park S., Hwang C.J., Lee D.-H., Kim N.Y., Nam H.W., Kang H.W., Lee C.S., Ok C.H., Cho J.H. (2024). Risk Factors of Revision Operation and Early Revision for Adjacent Segment Degeneration after Lumbar Fusion Surgery: A Case-Control Study. Spine J..

